# High-throughput profiling of metabolic responses to exogenous nutrients in *Synechocystis* sp. PCC 6803

**DOI:** 10.1128/msystems.00227-24

**Published:** 2024-03-27

**Authors:** Vilhelmiina Haavisto, Zachary Landry, Sammy Pontrelli

**Affiliations:** 1 Institute of Molecular Systems Biology, Department of Biology, ETH Zürich, Zürich, Switzerland; 2 Department of Civil, Environmental and Geomatic Engineering, Institute of Environmental Engineering, ETH Zürich, Zürich, Switzerland; University of Technology Sydney, Glebe, New South Wales, Australia

**Keywords:** mixotrophy, *Synechocystis*, metabolomics, environmental microbiology, metabolism, exometabolomics

## Abstract

Cyanobacteria fix carbon dioxide and release carbon-containing compounds into the wider ecosystem, yet they are sensitive to small metabolites that may impact their growth and physiology. Several cyanobacteria can grow mixotrophically, but we currently lack a molecular understanding of how specific nutrients may alter the compounds they release, limiting our knowledge of how environmental factors might impact primary producers and the ecosystems they support. In this study, we develop a high-throughput phytoplankton culturing platform and identify how the model cyanobacterium *Synechocystis* sp. PCC 6803 responds to nutrient supplementation. We assess growth responses to 32 nutrients at two concentrations, identifying 15 that are utilized mixotrophically. Seven nutrient sources significantly enhance growth, while 19 elicit negative growth responses at one or both concentrations. High-throughput exometabolomics indicates that oxidative stress limits *Synechocystis’* growth but may be alleviated by antioxidant metabolites. Furthermore, glucose and valine induce strong changes in metabolite exudation in a possible effort to correct pathway imbalances or maintain intracellular elemental ratios. This study sheds light on the flexibility and limits of cyanobacterial physiology and metabolism, as well as how primary production and trophic food webs may be modulated by exogenous nutrients.

**IMPORTANCE:**

Cyanobacteria capture and release carbon compounds to fuel microbial food webs, yet we lack a comprehensive understanding of how external nutrients modify their behavior and what they produce. We developed a high throughput culturing platform to evaluate how the model cyanobacterium *Synechocystis* sp. PCC 6803 responds to a broad panel of externally supplied nutrients. We found that growth may be enhanced by metabolites that protect against oxidative stress, and growth and exudate profiles are altered by metabolites that interfere with central carbon metabolism and elemental ratios. This work contributes a holistic perspective of the versatile response of *Synechocystis* to externally supplied nutrients, which may alter carbon flux into the wider ecosystem.

## INTRODUCTION

Cyanobacteria are primary producers found in virtually all terrestrial and aquatic ecosystems with access to light, where they carry out essential biogeochemical and ecological functions. Most importantly, they play a prominent role in global carbon fixation (1, 2), where they form the base of the food web by releasing carbon-containing compounds that support the growth of heterotrophic microbes in the surrounding environment (3, 4). However, cyanobacteria also respond sensitively to exogenous nutrients that may originate from anthropogenic or other biological sources (5–7). Changes to cyanobacterial physiology may have cascading effects on the composition and behavior of the heterotrophic bacterial community and the wider ecosystem, underscoring the importance of understanding how a broad range of exogenous nutrients affect cyanobacteria and other primary producers on a molecular level.

The effects of certain exogenous nutrients on cyanobacterial growth and population dynamics have been observed previously. For example, in aquatic ecosystems, eutrophication can spur large blooms as cyanobacteria take advantage of excess nitrogen and phosphorus (5), while chemical pollution can restructure entire communities by eliminating sensitive species and selecting for resistant ones (6, 7). Alternatively in the lab, cultures supplemented with carbon sources can either experience growth inhibition or grow mixotrophically, using auto- and heterotrophic modes of metabolism simultaneously (8, 9). Mixotrophy can be an important determinant of microbial fitness in many habitats (10, 11). For example, in aquatic environments, mixotrophic growth may enable the highly abundant marine cyanobacterium *Prochlorococcus* to survive at depths where light penetration is insufficient for them to survive on photoautotrophic growth alone (12).

Though growth and population-level responses have been characterized, we still lack a comprehensive understanding of how exogenous nutrients influence cyanobacterial exudation, which may have cascading effects as exuded compounds fuel heterotrophic microbes. These microbes not only carry out functions that are important for the broader ecosystem (13–18) but also engage in a bidirectional relationship with phototrophs, as they produce metabolic byproducts and micronutrients (19) or remineralized high molecular weight photosynthate (13, 20) that can, in turn, be used mixotrophically by cyanobacteria.

Cyanobacterial mixotrophic capabilities have thus far been demonstrated across phylogenetically disparate species, using carbon sources including acetate (21, 22), glycerol (8), and some amino acids (9) and monosaccharides (8). Yet, cyanobacteria encode a wide range of transporters for compounds including amino acids and oligopeptides (23) and urea and sugars (24), suggesting that the range of mixotrophically assimilable compounds may be wider than what has been shown for any individual species so far.

Here, we explore the mixotrophic capabilities of the model cyanobacterium *Synechocystis* sp. PCC 6803 (hereafter *Synechocystis*). We determine how supplementation of a broad range of metabolites alters its growth and exudate to understand the physiological processes that may be perturbed by exogenous nutrients. To overcome the inherent throughput limitations of typical, flask-based cyanobacterial cultivation, we developed a high throughput cyanobacterial culturing platform to systematically screen the growth and exudation behavior of *Synechocystis* supplemented with 32 metabolites at two different concentrations. We measured nutrient uptake and exudation using high-throughput exometabolomics, allowing us to identify cellular processes that define how *Synechocystis* responds to exogenous nutrients.

## RESULTS

### Development of a high-throughput cultivation system

Evaluating cyanobacterial responses to a wide range of growth conditions has historically been limited by cultivation throughput. Cyanobacteria, as well as other photoautotrophic microorganisms, are commonly cultured in flask-based setups (25–27) that are inherently low-throughput. One risk of using microtiter plates, which are routinely used for high-throughput culturing of microorganisms, is that insufficient gas exchange can reduce photoautotrophic growth via CO_2_ limitation (28). CO_2_ (26, 29, 30) can be supplemented by adding sodium bicarbonate directly to the culture medium (31); however, this can lead to alkalinization as the bicarbonate buffer equilibrates with atmospheric CO_2_, which may in turn inhibit cyanobacterial growth (32).

**Fig 1:**
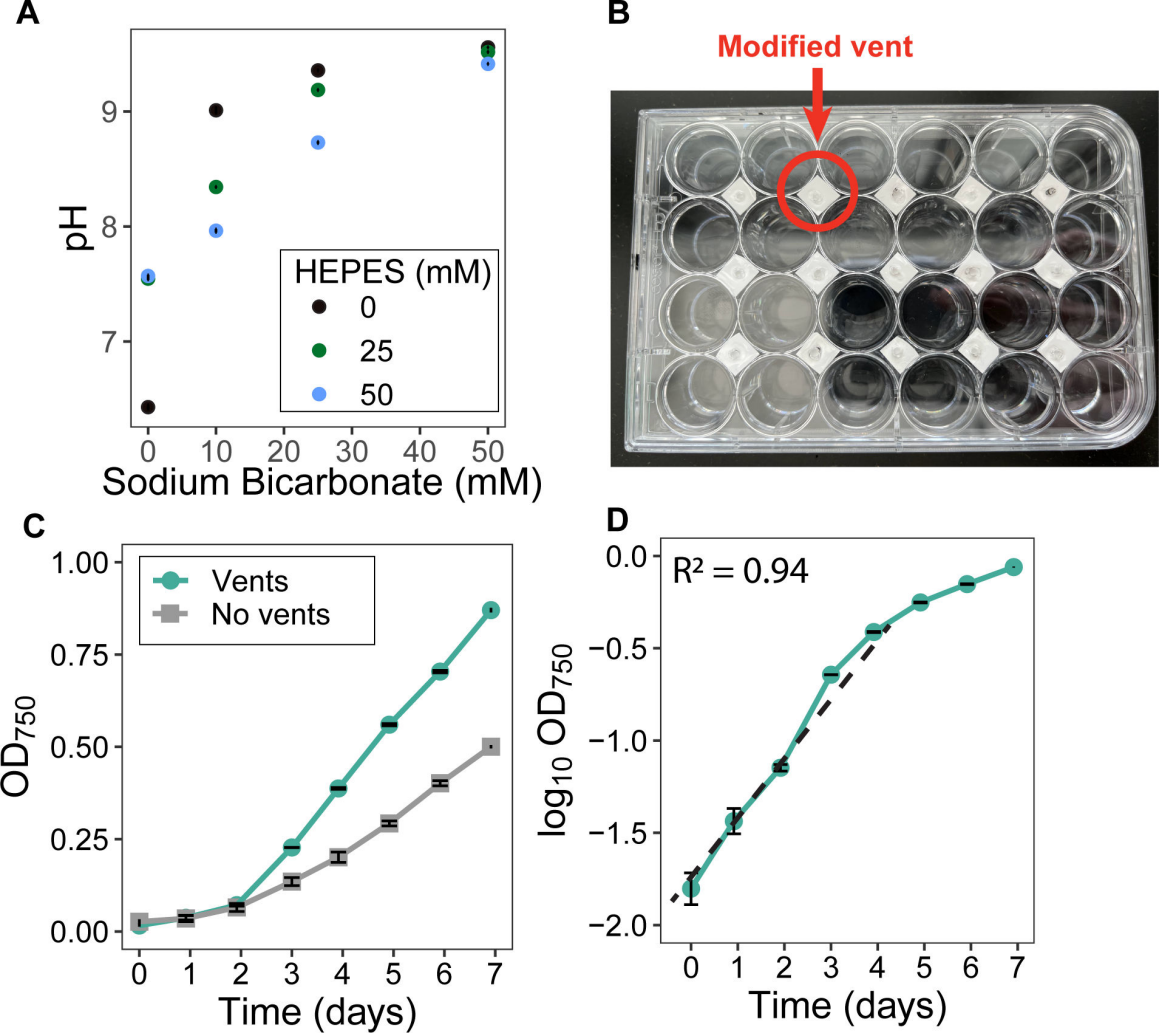
Growth of *Synechocystis* in an optimized high-throughput cultivation setup. All error bars represent SD around the mean. (**A**) Abiotic incubation of different concentrations of HEPES buffer and sodium bicarbonate in BG-11 medium. (**B**) Photograph of the modified 24-well plate lid on top of a commercially available 24-well plate. (**C**) OD_750_ growth curves of *Synechocystis* in 24-well plates with and without vented lids. (**D**) The vented lids allow *Synechocystis* to grow exponentially in the first 4 days. The dashed line indicates a linear regression between days 0 and 4, with the R^2^ indicated on the plot. All error bars represent the SD of the mean of three independent cultures.

One possibility to overcome such alkalinization is to buffer the medium. To test the feasibility of this approach, we performed an abiotic incubation of BG-11 medium supplemented with various concentrations of sodium bicarbonate and HEPES buffer. We observed that even low concentrations of sodium bicarbonate (10 mM) could not be buffered by the highest tested concentration of HEPES (50 mM) to a pH below 8 (Fig. 1A). Sodium bicarbonate is usually supplied at concentrations between 10 and 50 mM (33–35), and by decreasing the concentration below 10 mM to reduce alkalinization, we would likely lose its positive effect on growth. Meanwhile, further increasing HEPES concentrations could induce osmotic changes that would alter cyanobacterial physiology, which is also undesirable. Considering these limitations, we concluded that bicarbonate supplementation is not appropriate for our high-throughput culturing platform. Another option to mitigate CO_2_ limitation is gas-permeable microplate seals. However, these will block light and are impractical for cyanobacterial growth.

To overcome CO_2_ limitation, we modified a microtiter plate lid, placing vents between the wells for aeration, which were covered with a polyvinylidene difluoride (PVDF) membrane (pore size 0.45 µm) to prevent airborne contamination (Fig. 1B). These lids allowed us to achieve robust cyanobacterial growth in 24-well plates while relying on atmospheric CO_2_ concentrations (Fig. 1C and D), with exponential growth occurring in the first 4 days. We chose to use 24-well plates to maximize the volume available for sampling over longer experiments and to improve aeration in the wells. The plates also allowed us to measure cyanobacterial growth *in situ* using a plate reader.

Considering that bacterial contamination can compromise our experimental design, we performed a control experiment to test whether our culturing device can protect against bacterial contamination despite the vented lids (Fig. S1). We inoculated *Synechocystis* into our 24-well vented plates in monoculture or coculture with one of three environmental heterotrophic isolates. After 10 days of growth measurements, we measured the abundances of heterotrophs in all cultures using flow cytometry. We found no detectable heterotrophs in the monocultures, while heterotrophs grew to 5%–15% of the population after they were deliberately inoculated, demonstrating that our vented lids protect against bacterial contamination. Altogether, we developed and implemented a culturing platform that allowed us to expand the number of different growth conditions we can test at once.

### Growth response to metabolite supplementation

**Fig 2:**
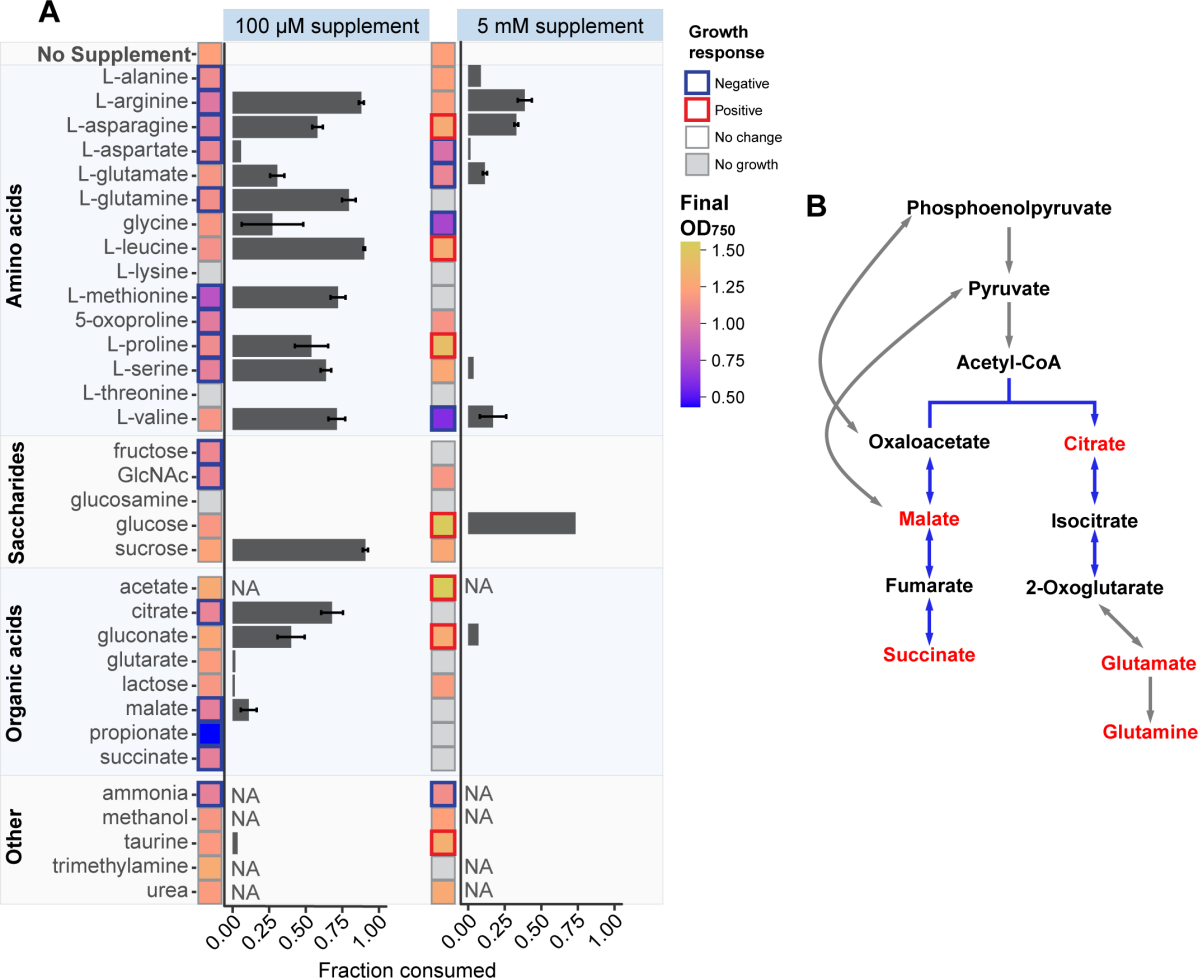
Growth and metabolite consumption. (**A**) Growth of *Synechocystis* after 10 days with supplementation of metabolites at 100 µM or 5 mM concentrations. Gray squares signify conditions where no growth is observed, and colors represent the final OD_750_ after 10 days. The border represents conditions where there is a significant (*P* < 0.05, Welch’s t test) increase (red) or decrease (blue) in OD_750_ compared to the no supplement control. Horizontal bars represent the fraction of the supplemented metabolite that is consumed after 10 days of growth. Compounds whose fraction consumed is marked “NA” are unable to be measured using the applied metabolomics method. All experiments were performed in triplicate, and error bars represent SD around the mean. (**B**) Diagram of the branched tricarboxylic acid (TCA) cycle in *Synechocystis* (blue lines) and related reactions. Metabolites in red inhibit growth when supplemented.

Using our high-throughput culturing platform, we tested how nutrient supplementation influences the growth of *Synechocystis* sp. PCC 6803. We grew *Synechocystis* under a 12:12 light:dark cycle and in the presence of one of 32 metabolites that represent a broad range of amino acids, organic acids, monosaccharides, and C1 compounds. We included compounds that have not previously been tested for consumption by *Synechocystis* during growth, as well as glucose and acetate, which have widely been recognized as mixotrophic substrates for (36, 37) *Synechocystis*. Of the chosen compounds, several have previously been shown to be incorporated into *Synechocystis* biomass using ^14^C labeled substrates (Table 1). However, these experiments typically measure uptake within seconds to minutes, which do not reflect whether the metabolite inhibits growth, can be used as a source of carbon or nitrogen to substantially improve biomass yield, or alters *Synechocystis’* exudation behavior. We supplemented metabolites at a high concentration of 5 mM and a lower concentration of 100 µM, yielding a total of 65 growth conditions including the non-supplemented control (8, 9, 22, 38). We monitored growth using OD_750_ (Fig. 2A) as a proxy for cyanobacterial abundance and measured metabolite consumption after 10 days of growth using liquid chromatography quadrupole time of flight mass spectrometry (LC-QTOF-MS).

**TABLE 1:** Metabolites that *Synechocystis* is known to consume, observed either in this or previous works^*a*^

Compound	Method	Reference
Sugars		
Glucose	Medium drawdown	This study (36)
Sucrose	Medium drawdown	This study
Organic acids		
Gluconate	Medium drawdown	This study
Malate	Medium drawdown	This study
Citrate	Medium drawdown	This study
Acetate	Medium drawdown	(37)
C1 compounds		
Urea	^14^C incorporation	(39)
Amino acids		
L-Alanine	^14^C incorporation	(38)
L-Arginine	^14^C incorporation	(38, 40)
	Medium drawdown	This study
L-Asparagine	^14^C incorporation	(38)
	Medium drawdown	This study
L-Glutamate	^14^C incorporation	(38)
	Medium drawdown	This study
L-Glutamine	^14^C incorporation	(38, 40)
	Medium drawdown	This study
Glycine	^14^C incorporation	(38)
	Medium drawdown	This study
L-Histidine	^14^C incorporation	(38, 40)
L-Leucine	^14^C incorporation	(38)
	Medium drawdown	This study
L-Lysine	^14^C incorporation	(38, 40)
L-Methionine	Medium drawdown	This study
L-Phenylalanine	^14^C incorporation	(38, 40^38^, ^40^)
L-Proline	^14^C incorporation	(38)
	Medium drawdown	This study
L-Serine	^14^C incorporation	(38)
	Medium drawdown	This study
L-Valine	Medium drawdown	This study

^*a*^ ^14^C incorporation refers to the uptake of isotopically labeled metabolites by *Synechocystis*, measured in seconds to minutes. Medium drawdown refers to measurements of metabolite depletion in the culture medium of *Synechocystis* after growth.

Of the 64 conditions with metabolite supplementation, seven significantly improved the growth of *Synechocystis* (*P* < 0.05, Welch’s *t* test), while 36 partially or completely inhibited growth. These growth effects were highly concentration-dependent; conditions with 5 mM metabolite supplementation comprise all cases with growth improvements and 12 of the 15 cases with complete growth inhibition. Due to limitations in the coverage of metabolites using mass spectrometry, we were unable to measure the consumption of urea, trimethylamine, methanol, ammonia, and acetate. However, *Synechocystis* consumed 15 of the remaining 27 metabolites, with mixotrophy here defined as a statistically significant (*P* < 0.05, Welch’s *t* test) reduction of at least 10% in measurement readout from the mass spectrometer in either the 5 mM or 100 µM supplementation condition (Fig. 2A). Mixotrophy and final yield appear to be uncoupled, as exemplified in 10 cases where *Synechocystis* reached a lower yield (OD_750_ after 10 days of growth) even when the metabolite was consumed, and three cases where *Synechocystis* reached a higher yield when no metabolite was consumed. While these results demonstrate the breadth of mixotrophic capabilities of *Synechocystis*, they also illustrate that growth limitations cannot solely be attributed to carbon or energy limitations and hint that exogenous nutrients may interfere with other physiological processes.

Of the seven metabolites that improved growth, an increase in cell density above 10% after 10 days of growth was observed with acetate, glucose, proline, and taurine. This reflects previous reports that *Synechocystis* uses glucose and acetate for mixotrophic growth, increasing its yield (11) (38). Proline has been recognized as an antioxidant whose intracellular concentration increases in cyanobacteria under high light conditions and has been shown to have positive effects against a large number of environmental stressors in higher plants, although its mechanisms are not entirely understood (41–43). Similarly, taurine has antioxidant properties against reactive oxygen species (ROS) in higher plants (44) which are also produced during cyanobacterial photosynthesis (45). Additionally, taurine represents a potential source of sulfur that can be used to fuel the production of glutathione, a vital metabolite for the mitigation of oxidative stress in (46) *Synechocystis*. Proline was partially consumed when supplied at 100 µM concentrations while taurine was not, and neither proline nor taurine was consumed when supplemented at 5 mM (Fig. 2A). These results suggest that the growth benefits of these metabolites may stem from their roles as antioxidants, not supplemental sources of carbon or nitrogen.

Supplementation of citrate, fructose, glutamine, glutarate, glutamate, succinate, malate, methionine, propionate, and threonine at 5 mM all resulted in no growth, while lysine, propionate, and threonine prevented growth even at 100 µM. The metabolites citrate, glutamine, glutamate, malate, and succinate are all involved in the tricarboxylic acid (TCA) cycle or are close derivatives of TCA intermediates (Fig. 2B) (47, 48). This suggests that supplementing TCA cycle-related metabolites may hinder its function and growth.

### Condition-specific metabolite exudation

We performed untargeted metabolomics analysis on the cell-free culture supernatants to determine how the exudation of *Synechocystis* changes in response to metabolite supplementation. Overall, we detected 925 ions, of which 62 could be annotated based on exact mass that was found only after 10 days. We then normalized each metabolite’s intensity across all conditions, allowing us to compare changes in the relative concentrations of each metabolite (Data Set S1).

**Fig 3:**
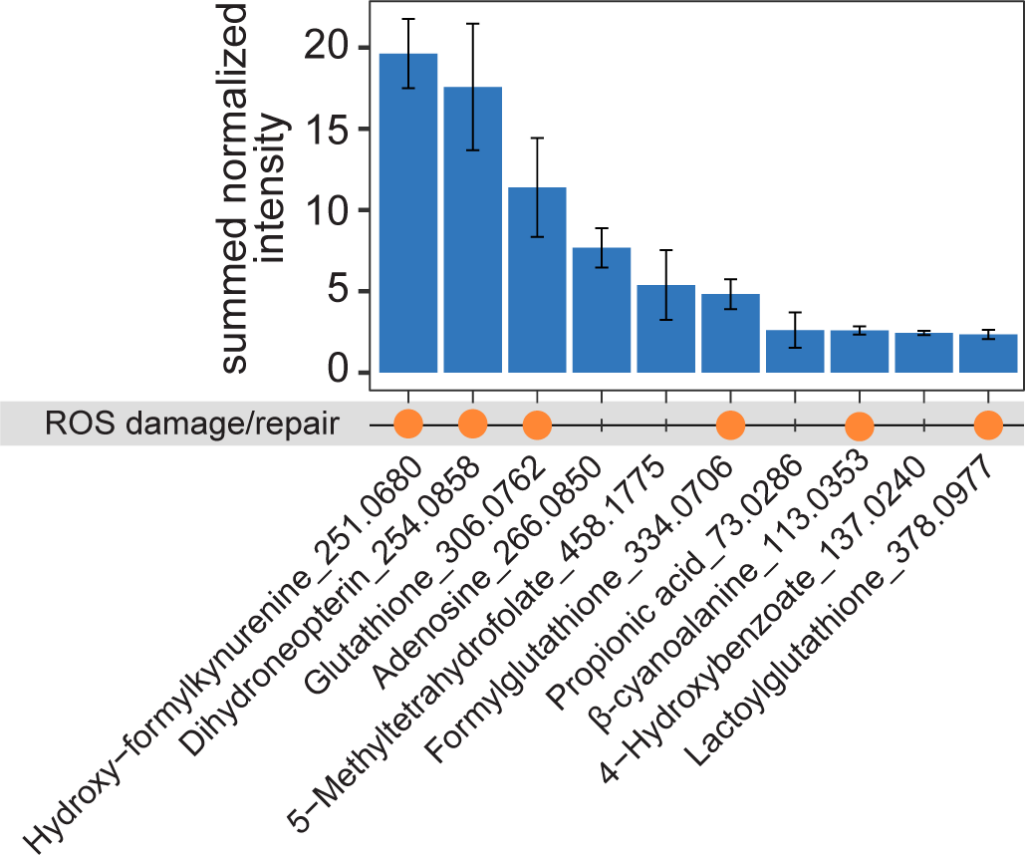
Most frequently produced metabolites across all conditions. Ten metabolites with the highest summed normalized intensity. Orange dots signify that these metabolites are involved in ROS damage/repair. For each annotated metabolite, the mass-to-charge ratio (*m*/*z*) is noted. All experiments are performed in triplicate, and error bars represent SD around the mean.

We identified which metabolites are produced most frequently by summing the normalized intensities of each metabolite across all conditions and ranking each metabolite according to its summed normalized intensity. Of the 10 metabolites with the highest rank, five have the capacity to mitigate oxidative stress via ROS scavenging (Fig. 3), including dihydroneopterin, glutathione, formylglutathione, lactoylglutathione, and β-cyanoalanine (49–51). Additionally, hydroxy-formykynurenine, the most frequently produced metabolite (produced in 44 of the 50 cultures with a visible OD_750_), is one of several compounds that cyanobacteria and other photoautotrophic organisms produce upon oxidative damage to photosystem II (PSII) (52, 53). The formation and metabolic response to ROS may thus be crucial for *Synechocystis* growth under our conditions

**Fig 4:**
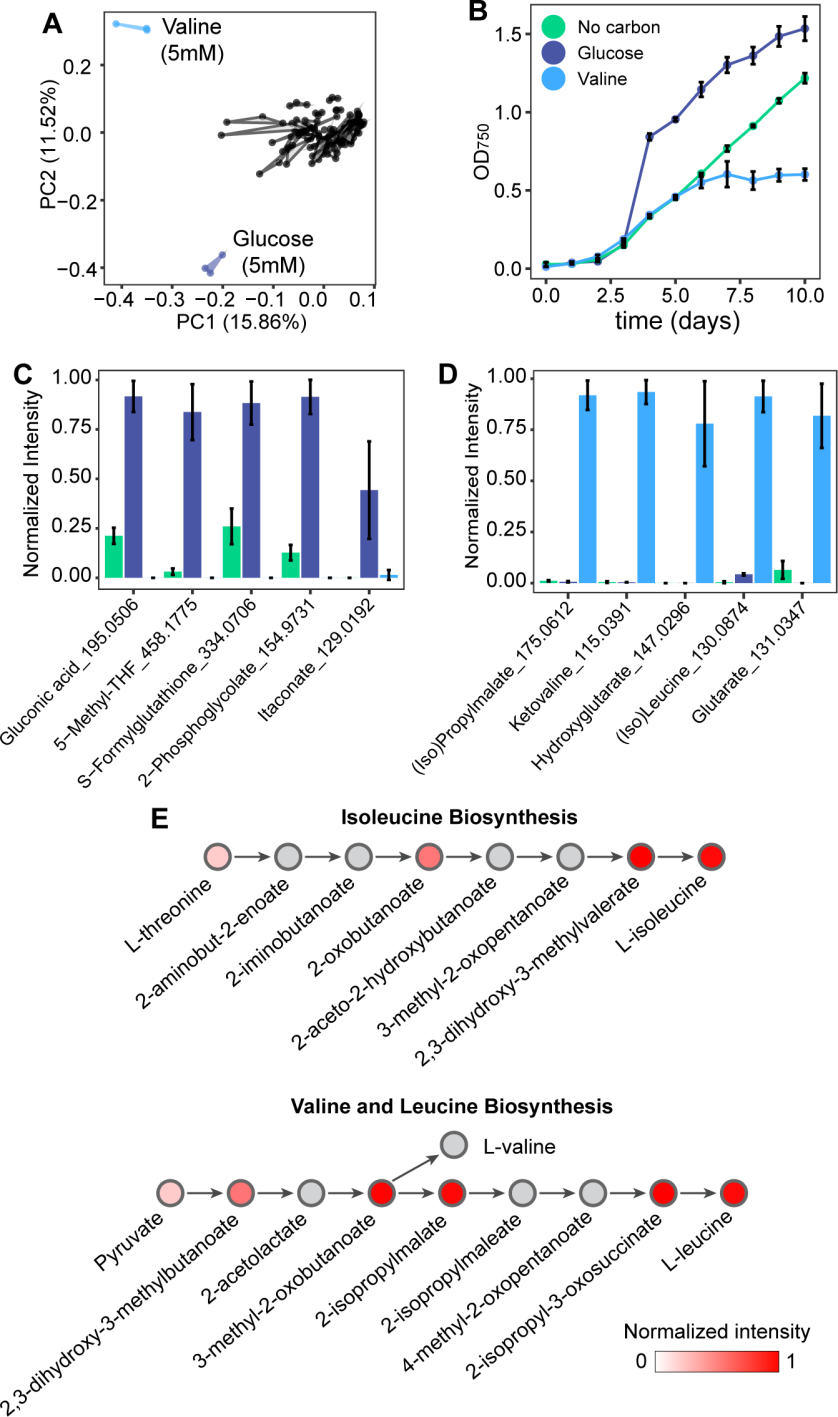
Condition-specific metabolite exudation. (**A**) PCA of all exometabolite profiles in all growth conditions. (**B**) Growth curve of *Synechocystis* with or without 5 mM supplementation of glucose or valine. Metabolites with the (C) most negative or (D) most positive loading on the second principal component (PC2), and their normalized intensities for *Synechocystis* growth with 5 mM glucose (dark blue), 5 mM valine (light blue), or no supplementation (green). (**E**) Isoleucine, valine, and leucine biosynthesis pathways. The color of the circle represents the normalized intensities of metabolites produced with 5 mM valine supplementation. Gray circles were not measured in our metabolomics data set. Experiments were performed in triplicate, and error bars represent the SD of the mean. Abbreviation: 5-methyltetrahydrofolate; 6-Methyl-THF.

We next examined changes in exuded metabolites, collectively referred to as the exometabolome, specific to each supplemented metabolite. To this end, we performed principal component analysis (PCA) on the exometabolome profiles of *Synechocystis* in each growth condition (Fig. 4A; Fig. S2). The PCA revealed a clear separation of glucose and valine (supplemented at 5 mM) from the other culture conditions in opposite directions along PC2. Glucose and valine exerted positive and negative influences on the growth of *Synechocystis*, respectively (Fig. 4B), suggesting that differences in the exometabolome profiles are due to distinct underlying physiological responses. To investigate these responses, we identified which metabolites contribute to the separation observed in the PCA. Of metabolites that have the most positive or negative loading on PC1, most failed to show a clear separation between the valine and glucose conditions (Fig. S3 and S4). However, glucose and valine exometabolome profiles were clearly distinguished along PC2; therefore, we identified five metabolites with the most positive or negative loading on this component (Fig. 4C and D). Gluconate, 5-methyltetrahydrofolate, formylglutathione, 2-phosphoglycolate, and itaconate, which have the most negative loading, were produced at high relative abundances under 5 mM glucose supplementation and at higher concentrations than any other condition (Fig. S5). The five metabolites exhibiting the most positive loading on the second principal component are produced under 5 mM valine supplementation and no other condition: (iso)propylmalate, ketovaline, hydroxyglutarate, (iso)leucine, and glutarate (Fig. 4D).

The exometabolome profile observed under valine supplementation suggests perturbations to amino acid metabolism, as all five metabolites that contribute to the separation of valine in the PCA are involved in either amino acid biosynthesis or degradation pathways. In addition, valine supplementation, similar to other tested amino acids, inhibits the growth of *Synechocystis* (Fig. 2A and 4B) (9, 54–57). To further explore the effects of valine supplementation on *Synechocystis*, we performed a metabolic pathway ranking to identify which pathways are most represented by the exuded metabolites (see Materials and Methods). Branched-chain amino acid biosynthesis is the highest ranked (Table S1), as we measured 11 metabolites exuded after valine supplementation that are involved in this metabolic pathway (Fig. 4E). These findings provide evidence for the hypothesis that the reduced growth conferred by high valine concentrations, and the resulting exudation of these metabolites may be an adaptation to maintain optimal intracellular concentrations of amino acids or to maintain an optimal C:N ratio.

## DISCUSSION

Despite the critical role of cyanobacteria as primary producers across different ecosystems, we lack a comprehensive analysis gauging both their growth and exudation response to a broad range of metabolites. Using a high-throughput cultivation platform, we were able to assess the responses of *Synechocystis* sp. PCC 6803 to a large panel of supplemented metabolites, uncover how these metabolites change growth and exudation behavior, and identify previously unknown metabolites potentially used for mixotrophic growth.

We screened through 32 metabolites at two concentrations and found that at least 15 metabolites were consumed mixotrophically. This includes six previously unreported metabolites: sucrose, malate, citrate, gluconate, methionine, and valine. Moreover, our measurements of metabolites that are drawn down in the growth medium are mostly consistent with previous reports of ^14^C metabolite uptake. Two inconsistencies are alanine and lysine, which were not consumed in our experiments but have previously been shown to be incorporated into *Synechocystis* biomass (38, 58). Here, we find that lysine causes complete growth inhibition, even at 100 µM concentrations. Previous results have demonstrated that *Synechocystis* encodes non-specific amino acid transporters. Such transporters may be maintained due to their multifunctionality; for example, cyanobacteria such as *Microcystis aeruginosa* may have evolved to maintain the lysine uptake system despite its toxicity because it can also be used to import other, non-toxic amino acids, such as arginine and ornithine. Complementary to these findings, our results suggest that although *Synechocystis* may encode active transporters for a given metabolite—in this case, lysine—this metabolite may otherwise be inhibitory and is not.

Supplementation most often inhibited, rather than enhanced, cyanobacterial growth. In particular, supplementation with TCA cycle intermediates or closely related compounds provided a high frequency of partial or complete growth inhibition at 5 mM concentrations. Disruptions to the bifurcated TCA cycle of *Synechocystis* could stem from several sources. In *Synechocystis*, phosphoenolpyruvate from autotrophic carbon fixation enters the TCA cycle via conversion to oxaloacetate. The malate dehydrogenase enzyme in *Synechocystis* has been shown to directionally favor the conversion of oxaloacetate to malate, rather than the oxidative direction of malate to oxaloacetate, at ~361:1 (59). Threonine, lysine, and methionine are all synthesized from oxaloacetate. These compounds may represent a downstream sink for oxaloacetate, and supplementation may induce regulatory changes or product inhibition that necessitates the redirection of carbon flux to succinate, which would eventually accumulate or need to be assimilated via alternative pathways (60). Similarly, glutamine is normally produced from 2-oxoglutarate and may represent a sink for 2-oxoglutarate. The presence of exogenously added glutamine would also likely disrupt this sink, resulting in the stagnation of the flow of this branch of the TCA cycle. These metabolites may also directly interfere with the activity of TCA cycle enzymes. For example, citrate and succinate have been shown to cause significant reductions in fumarase activity (61), while citrate and malate reduce the activity of phosphoenolpyruvate carboxylase (62). Meanwhile, high concentrations of propionate are toxic to *Synechocystis* and other cyanobacteria (63), possibly via shrinking of the intracellular acetyl-CoA pool (64). Collectively, the supplementation of central metabolites may cause disruptions to the TCA cycle and thus inhibit the growth of *Synechocystis*.

Interestingly, some metabolites, such as asparagine and proline, show a growth enhancement at 5 mM but inhibit growth at 100 µM. This effect could be attributed to their potential deleterious impact on growth. For instance, asparagine and proline induce negative feedback inhibition on nitrogenase activity in Cyanobacteria *Anabaena cylindrica* PCC 7122 and *Gloeocapsa sp*., which may interfere with nitrogen metabolism. At higher concentrations, however, any negative regulatory effects might be counteracted by beneficial properties such as ROS scavenging, as indicated by our findings with proline. This phenomenon mirrors observations with quinolone antibiotics, such as nalidixic acid, where lethality is also inversely correlated with concentration due to the higher concentrations that prevent ROS formation.

In most of our growth conditions, even without metabolite supplementation, *Synechocystis* produced hydroxy-formykynurenine, a metabolite formed as a consequence of oxidative damage in PSII (52, 53). Additionally, 5 out of the 10 most frequently produced metabolites are engaged in ROS scavenging, indicating an adaptive response of (45) *Synechocystis* to counteract oxidative stress. Concurrently, we found that supplementation at 5 mM of two metabolites with known antioxidant properties, proline and taurine (39, 40, 42, 65), significantly improved growth. This suggests that ROS damage and repair are growth-limiting factors for *Synechocystis* and may be alleviated by exogenous compounds with antioxidant properties. Oxidative stress occurs as a normal part of photosynthesis, particularly if light-driven electron transport proceeds faster than CO_2_ fixation, which consumes these electrons. This may be an indicator that our light conditions were too strong and caused the formation of ROS. However, we chose light conditions (70 µmol m^−2^ s^−1^) that fall within the range normally used for laboratory cultivations (50–100 µmol m^−2^ s^−1^) to be consistent with other works. Nevertheless, an area of further study may be to investigate the effect of light intensity on the metabolite secretion of *Synechocystis*.

We explored how metabolite supplementation changes the exudation behavior of *Synechocystis* and identified two conditions, glucose and valine supplied at 5 mM, where the exometabolome diverged compared to the other conditions. One metabolite exuded during glucose supplementation is gluconate, which can be formed from the direct oxidation of glucose or via the spontaneous hydrolysis of the oxidative pentose phosphate pathway (oPPP) intermediate, glucono-1,5-lactone. *Synechocystis* relies on the oPPP as a high-flux route for sugar catabolism (66), as well as other marine cyanobacteria (67), meaning that gluconate may be produced as an overflow product or unwanted side product as a consequence (66, 68). A similar observation has been made in the Alphaproteobacterium *Gluconobacter oxydans*, wherein high glucose concentrations not only induce gluconate production but also prevent its uptake (69). Additionally, 2-phosphoglycolate is produced by ribulose 1,5-bisphosphate carboxylase (RuBisCo) as an unwanted by-product of photorespiration, where oxygen competes with carbon dioxide for the desired carboxylation reaction in photosynthesis (70). Because glucose increases the yield of *Synechocystis*, which will inherently have a higher demand for CO_2_, increased photorespiration and 2-phosphoglycolate concentrations under glucose supplementation may be a consequence of a depleted CO_2_ supply. However, it is unclear why only glucose supplementation resulted in the production of 2-phosphoglycolate compared to other metabolites that also significantly improved growth, such as acetate, asparagine, and gluconate. A probable explanation is that glucose supplementation led to the highest cell density of *Synechocystis* (OD_750_ 1.53 compared to the next highest cell density of proline, OD_750_ of 1.43). This likely intensified CO_2_ limitations.

In contrast to glucose, valine significantly impaired the growth of *Synechocystis* when supplied at 5 mM. We identified that the exuded metabolites overwhelmingly represented pathways including branched-chain amino acids and their precursors, as well as several other amino acid degradation products. Amino acid toxicity has been observed in *Synechocystis* and other cyanobacteria, as well as chemoautotrophic and methylotrophic bacteria, and has been attributed to a stoichiometric imbalance in amino acid metabolism. The exudation of these metabolites might indicate an attempt by *Synechocystis* to restore its intracellular amino acid balance or C:N ratio. However, supplementation of other branched-chain amino acids did not have the same effect—in fact, (iso)leucine supplementation at 5 mM significantly improved the growth of *Synechocystis*, although it was not consumed. Other amino acids also did not affect the exometabolome in this way, although many were consumed to a similar degree and also negatively affected growth. One explanation for this observation could be that valine alters the regulation of branched-chain amino acid biosynthesis, as it has been shown in other organisms that valine specifically causes a feedback inhibition on acetohydroxyacid synthase (71). However, further investigation of the regulatory influence of valine in *Synechocystis* must be performed to better understand this observation.

Using high-throughput metabolomics, we were able to measure the consumption and exudation of low molecular weight, polar metabolites, including amino acids, sugars, and organic acids; however, we did not measure extracellular polymeric substances (EPS), which are important high molecular weight component of cyanobacterial exudates (20). EPS can also provide carbon to heterotrophic bacterial populations (13, 20, 72) and is important for the colony-forming lifestyles of certain cyanobacterial strains (73). Recent work has shown that the composition of *Synechocystis* EPS changes with environmental nutrient conditions (74, 75), but to our knowledge, no screen like that presented here has been conducted with EPS quantity or composition as a readout. Furthermore, the ecological functions of extracellular vesicles in cyanobacteria are only beginning to be explored (76), though they also transport metabolites and other cellular components into the extracellular environment. Work involving the broader exuded resources of cyanobacteria will be important for completing our understanding of their ecologically relevant responses to diverse nutrient conditions.

A limitation of this study is that the chosen concentrations of 5 mM and 100 µM are generally higher than typical concentrations found in the environment, which range from nanomole to low micromole concentrations of total dissolved free amino acids (77). These concentrations were chosen to broadly cover the large range of metabolite concentrations that have been supplemented to *Synechocystis* or other cyanobacteria in previous works. However, some of the metabolic responses observed here may fall outside responses that *Synechocystis* may have in natural environments. As an example, when *Synechocystis* is exposed to higher concentrations of metabolites, it could trigger their uptake by non-specific transporters that might otherwise restrict their absorption under lower concentrations. This could explain why we observed inhibition by high concentrations of central carbon metabolites. On the other hand, the chosen concentrations allow our work to assess mixotrophy by looking for increased growth yields, as has been observed in other works and here (Fig. 2 and 4B), and allow us to stay within concentration ranges where we can detect their consumption using high throughput metabolomics. By comparing the metabolic responses of *Synechocystis* to the broad range of metabolites, this still allows us to learn about *Synechocystis’* metabolism, its mixotrophic capabilities, and how it can adapt to different nutrient conditions.

With our high-throughput culturing platform, we were able to provide insight into how cyanobacterial physiology and metabolism respond to a large range of metabolites. This platform could be leveraged for other applications involving the cultivation of photoautotrophs under a large set of conditions, expanding the possibilities of research questions and feasible experimental setups. This could involve fields such as toxicology, where it is of interest to understand how cyanobacteria and algae respond to an ever-growing range of xenobiotics and other environmental pollutants, or microbial ecology, where the interactions among photoautotrophs and other microbes could be studied in a high-throughput manner.

## MATERIALS AND METHODS

### Strains

Axenic *Synechocystis* sp. PCC 6803 was obtained from the Pasteur Culture Collection (Paris, France). Heterotrophs *Acidovorax* sp., *Piscinibacter* sp., and *Staphylococcus* sp. were isolated from Switzerland’s Lake Greifensee (latitude: 47 36 43, longitude: 08 67 48). The resulting phototroph-heterotroph communities were streaked onto R2A agar plates, and single colonies were picked, re-grown in liquid R2A media, and preserved in glycerol stocks.

### Chemicals and culturing conditions

Axenic *Synechocystis* sp. PCC 6803 were maintained in 100 mL flasks by transferring to fresh media weekly. Cultures were verified axenic by streaking them onto a nonselective R2A medium and testing for colony-forming units after incubating at 30°C in the dark or by testing for the presence of heterotrophs with flow cytometry (Fig. S1). The medium for precultures was BG-11 medium (Sigma) supplemented with 25 mM HEPES sodium salt, adjusted to pH 7.5. For the screen, we used BG-11 composed of three stock solutions, supplemented with trace metals and sodium nitrate, with the addition of 25 mM HEPES sodium salt. The three stock solutions were prepared as follows: 0.1 g/L magnesium disodium EDTA, 0.6 g/L ferric ammonium citrate, 0.6 g/L citric acid monohydrate, and 3.6 g/L calcium chloride dihydrate (stock solution 1); 7.5 g/L magnesium sulfate heptahydrate (stock solution 2); 3.05 g/L potassium phosphate anhydrous (stock solution 3). The trace metal solution was made as follows: 2.86 g/L boric acid, 1.81 g/L manganese chloride tetrahydrate, 0.22 g/L zinc sulfate heptahydrate, 0.05 g/L copper sulfate anhydrous, 0.05 g/L cobalt(II) chloride hexahydrate, and 0.39 g/L sodium molybdate dihydrate. The stocks were combined as follows: for 1 L of medium, 10 mL each stocks 1–3, 1 mL trace metals stock, 18 mL 1M sodium nitrate stock, 100 mL 250 mM HEPES, and 851 mL sterile ddH_2_O. The stock, trace metals, HEPES, and sodium nitrate solutions were all filter sterilized (0.22 µM) and stored at 4°C prior to use. BG-11 media was made fresh for each preculture transfer or experiment. 0.5 M stock solutions of all supplemented metabolites were prepared in ddH_2_O, and filter sterilized (0.22 µM). The stocks were stored at 4°C and diluted accordingly into fresh BG-11 media for each experiment. Unless otherwise noted, all chemicals were obtained from Sigma Aldrich. All growth experiments were conducted at 30°C, with a light intensity of 70 µmol m^−2^ s^−1^ supplied by four neutral white LED modules (Waltron) and a 12:12 light:dark cycle. Cultures were continuously shaken at 220 rpm (100 mL flasks) or 400 rpm (24-well plates) with an orbital shaker (VWR) placed inside the incubator. Cyanobacterial growth was quantified by measuring optical density at 750 nm (OD_750_) using a Tecan Infinite Nano microplate reader (Tecan).

For the metabolite supplementation experiments, cultures in 24-well plates were inoculated from precultures to an OD_750_ of 0.01 and with a starting volume of 1.6 mL. Each metabolite at each concentration had three biological replicate cultures. To account for evaporation (approximately 60 µL per day per well, data not shown) and loss of volume due to metabolomics sampling, we replenished all wells with 240 µL water on day 4 and 180 µL on day 7. We also replenished the 100 µL that was sampled for metabolomics on day 5.

### Vented lids

To enable robust photoautotrophic growth in 24-well plates, custom-made vented lids were used to increase aeration in the wells. To make the lids, holes were made in the space between the condensation rings of a polystyrene 24-well plate lid (Corning) with an 18G hypodermic needle heated using a Bunsen burner. The holes were covered by attaching a square of PVDF filtration membrane (0.45 µm pore size, Millipore) on the inner face of the lid using clear PVA glue (UHU brand) to prevent airborne contamination during cultivation. The holes were positioned between, rather than on top of, the wells to avoid shading the cultures from the overhead lights in the incubator. Before use, the lids were wrapped in a single layer of cling film and sterilized by exposure to 254 nm UV radiation for 15 minutes inside a Spectrolinker XL-1500 UV-crosslinker (Spectro-UV). The lids were then placed onto pre-sterile 24-well plates in a sterile hood after the plates had been inoculated with cultures. The edges of the plates were sealed with Parafilm to prevent excess evaporation.

### Exometabolomics

For sample collection, 100 µL of culture was added to a 96-well v-bottom plate and centrifuged at 2,500 rcf for 20 minutes. The supernatant was collected and stored at −20°C until measurement. The sample was diluted 10-fold and measured using LC-QTOF-MS. Measurements were performed using an Agilent 6520 Time of Flight Quadrupole Time of Flight Mass Spectrometer in negative mode, high-resolution mode with a 0.9 Hz scan rate, and an acquisition mass range of 50–1,700 *m*/*z*. The drying gas was set to 10 L/min, the nebulizer to 30 psig, and the gas temperature to 325°C. Using an Agilent 1100 series liquid chromatography stack, 3 µL of sample was injected into an Agilent EC-CN Poroshell column (2.7 µm, 50 × 2.1 mm) using an adapted salt tolerant method to mitigate ion suppression caused by salts in the medium (78). The sample injection order was randomized, and all samples were injected in single technical replicates. The column was kept at 20°C, and the flow rate was 350 µL/min. The buffer used was 10% Acetonitrile (CHROMASOLV), 90% mass spectrometry grade water, and 0.01% Formic acid. The method was operated isocratically, and for every 40 samples, the column was washed by flushing for 5 minutes with 90% acetonitrile (CHROMASOLV), 10% mass spectrometry grade water, and 0.01% formic acid, followed by equilibration for 10 minutes with the isocratic buffer. Raw data for all measurements were subjected to a spectral processing and alignment pipeline using Matlab (The Mathworks, Natick, MA) as described previously (79). Peaks were annotated based on exact mass by matching them to metabolites predicted to partake in the *Synechocystis* metabolic network according to the Biocyc Database (80).

To compare metabolite production between culture conditions, all produced metabolites were normalized between 0 and 1 (Data Set S1). First, we calculated a limit of detection for each metabolite. This is the average metabolite intensity for BG-11 medium background sample, plus three times the SD of these background intensities. Any metabolite intensity that was below the limit of detection was reassigned to have the same intensity as the background medium. To remove the influence of supplemented metabolites on the normalization, for samples that have a supplemented metabolite, the intensity of this metabolite was reassigned to have the same intensity as the background medium. Next, all metabolite intensities were normalized between 0 and 1, where 0 is the intensity of the background medium and 1 is the intensity of the sample that has the highest concentration of the metabolite.

### Metabolic pathway ranking

Using all annotated metabolites that were detected in any culture condition, we identified which metabolic pathways they participate in based on the Biocyc pathway database of *Synechocystis* sp. PCC 6803. This yielded the total number of detected metabolites for each metabolic pathway. We then calculated the number of metabolites found in each metabolic pathway separately for each of our 65 culture conditions. Then, we scored each metabolic pathway’s importance in each culture condition by dividing the number of produced metabolites in the pathway by the total of detected metabolites across all pathways in that condition. We then ranked the pathways by this score to identify which pathways are most represented by the metabolites exuded in each culture condition.

### Testing for heterotrophs present in *Synechocystis* cultures

Heterotrophic bacterial isolates *Acidovorax* sp., *Pischinibacter* sp., and *Staphylococcus* sp. were precultured in R2A medium from single colonies overnight. Cultures were centrifuged, and the pellet was washed in fresh BG11 medium. These heterotrophs were then co-inoculated with *Synechocystis* in BG11 medium, where the heterotrophs were inoculated 1:10,000 from their preculture into 24-well plates with vented lids. *Synechocystis* was inoculated axenically in 12 additional wells. Cultures were grown for 10 days, growth was measured with OD_600_, and the culture medium was sampled and replenished at days 4 and 7, as described above. After 10 days, the percentage of the heterotroph population was measured using flow cytometry. Cultures were diluted 1:100 in 200 µL of BG11 with 1:5,000 SYTO40. After 20 minutes, samples were diluted 1:10 again into BG11 medium. A BD Fortessa II flow cytometer was used to count cells using the B690 filter (chlorophyl autofluorescence) and V450 filter (SYTO40). Events were plotted against these axes to separate signal from the cyanobacteria and heterotrophs.

## Data Availability

Raw spectral files have been deposited to the MassIVE database under accession no. MSV000093130.

## SUPPLEMENTAL MATERIAL

Data Set S1 msystems.00227-24-s0001.xlsx Normalized intensities and isomers.

Supplemental Material msystems.00227-24-s0002.docx Table S1; Figures S1-S5.

ASM does not own the copyrights to Supplemental Material that may be linked to, or accessed through, an article. The authors have granted ASM a non-exclusive, world-wide license to publish the Supplemental Material files. Please contact the corresponding author directly for reuse.
