# Supplementary material for: High-throughput profiling of metabolic responses to exogenous nutrients in Synechocystis sp. PCC 6803
Source: mSystems. 2024 Mar 27;9(4):e00227-24. doi: 10.1128/msystems.00227-24 (PMC11019784; doi:10.1128/msystems.00227-24)
Supplement: Supplemental Material — Table S1; Figures S1-S5. [file msystems.00227-24-s0002.docx]

**Supplementary information**

| Pathway | Exuded  compounds | Measurable  compounds | Pathway score |
| --- | --- | --- | --- |
| Superpathway of branched chain amino acid biosynthesis | 11 | 13 | 0.85 |
| L-isoleucine biosynthesis I (from threonine) | 5 | 6 | 0.83 |
| L-leucine biosynthesis | 5 | 6 | 0.83 |
| L-isoleucine biosynthesis II | 6 | 8 | 0.75 |
| superpathway of L-isoleucine biosynthesis I | 6 | 8 | 0.75 |
| L-threonine degradation I | 2 | 3 | 0.67 |
| L-valine biosynthesis | 3 | 5 | 0.60 |
| L-arginine degradation VI (arginase 2 pathway) | 2 | 4 | 0.50 |
| superpathway of L-threonine biosynthesis | 2 | 4 | 0.50 |
| superpathway of thiamine diphosphate biosynthesis II | 2 | 4 | 0.50 |

**Supplementary table 1:**

Metabolic pathways with the highest rank when *Synechocystis* is grown with 5 mM valine, based on the exometabolome profile. Measurable compounds are the total number of compounds in the pathway that were measurable in any growth condition. Exuded compounds are the number of compounds in the pathway that were exuded in the 5 mM valine condition. The pathway score is the percentage of measurable compounds that were exuded, and all pathways were ranked based on this score.


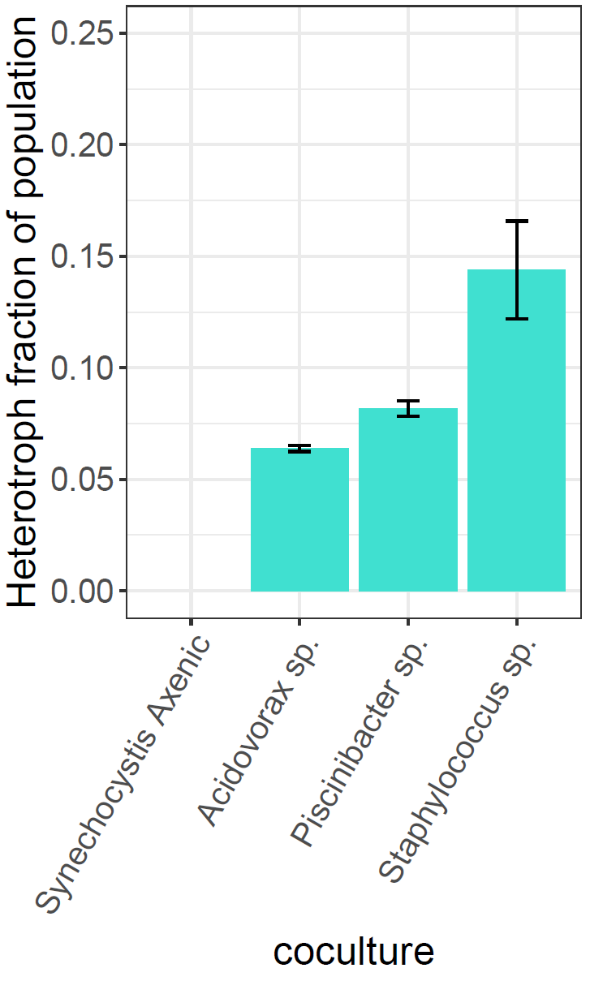
**Supplementary figure 1:** Heterotrophic bacteria counted in cultures of *Synechocystis* grown axenically (n=12) or co-inoculated (n=3) with one of three heterotrophic bacteria in 24 well plates with vented lids after 10 days. The fraction of the population that is heterotrophic was measured using flow cytometry. No heterotrophs were detected in the axenic control. Error bars represent standard deviation of the mean.


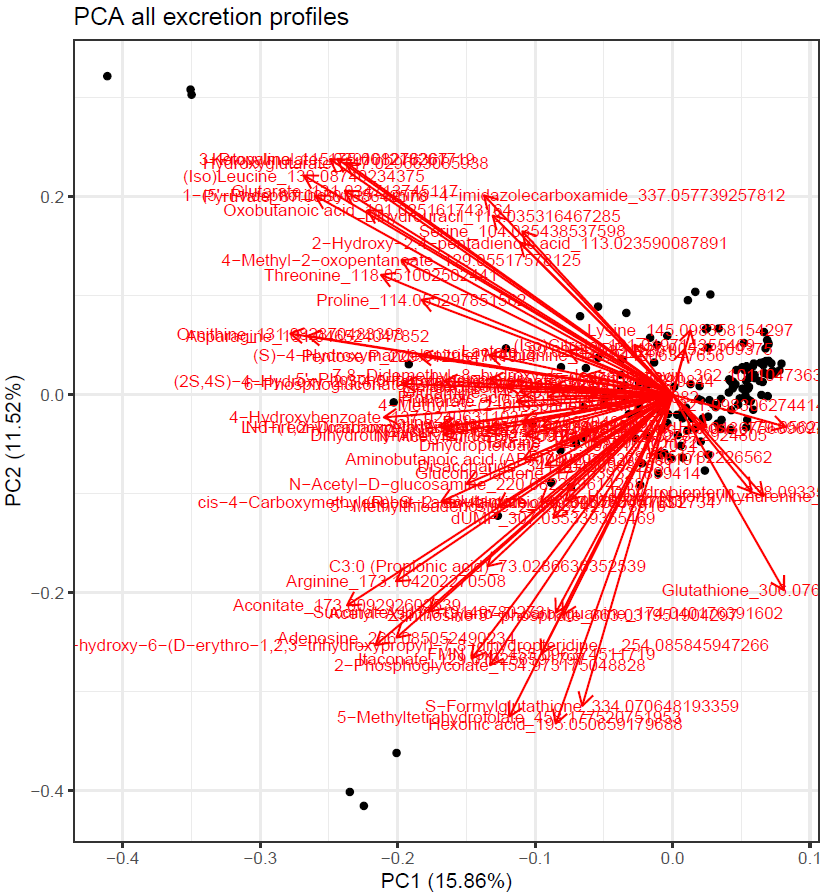
**Supplementary figure 2:** PCA loading plot of all secreted metabolite profiles.

**
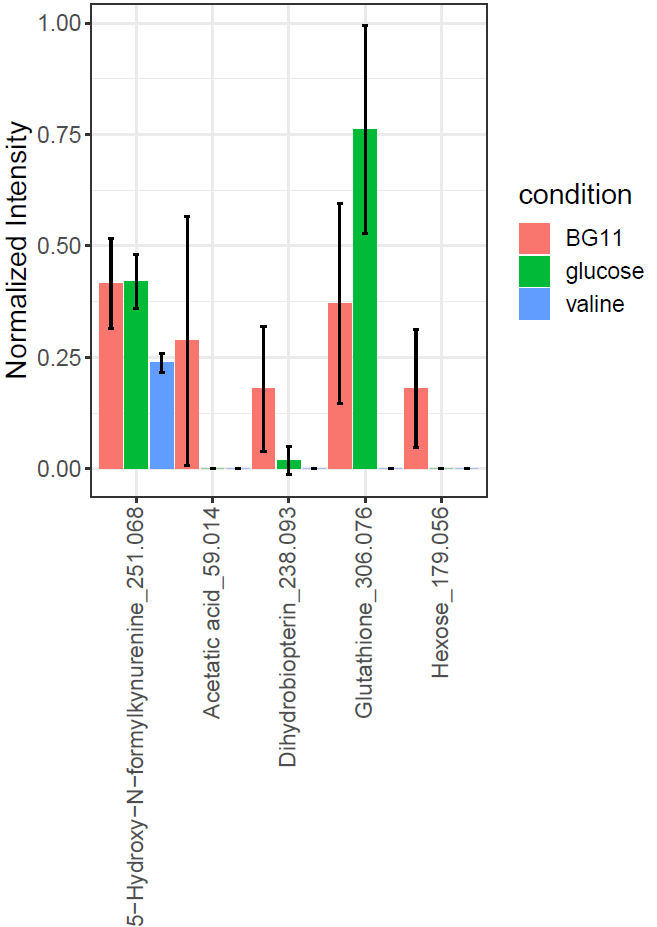
**

**Supplementary figure 3:** Normalized intensity of metabolites that have the most positive loading on the first principal component. Error bars represent standard deviation of the mean.

**
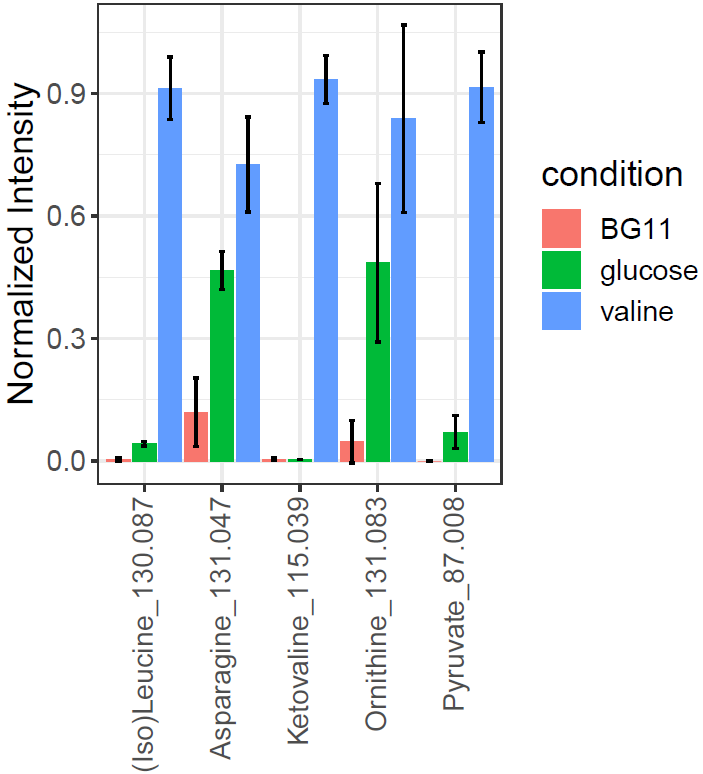
**

**Supplementary figure 4:** Normalized intensity of metabolites that have the most negative loading on the first principal component. Error bars represent standard deviation of the mean.


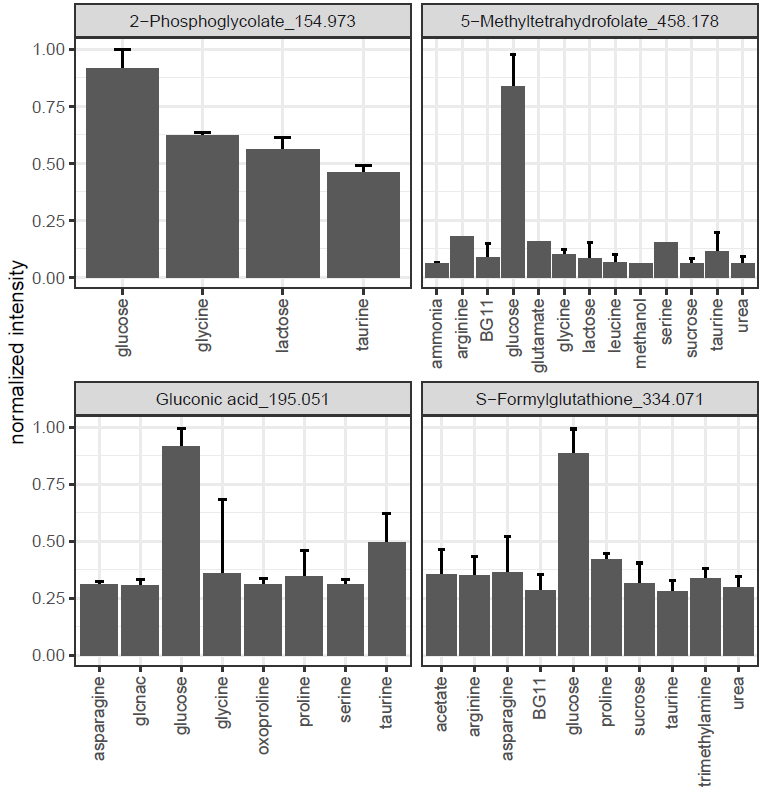


**Supplementary figure 5:** Metabolite concentrations of gluconate, S-Formylglutathione, 2-Phosphoglycolate and 5-methyltetrahydrofolate, which contribute most to the negative loading on the second component of PCA. All conditions in which the metabolite is detected above the limit of detection are listed. Not shown is itaconic acid, which is only detected with 5mM glucose supplementation. Error bars represent standard deviation of the mean of three replicate cultures.
